# Basic and clinical immunology – 3016. Successful desensitization to oxaliplatin for metastic colorectal carcinoma

**DOI:** 10.1186/1939-4551-6-S1-P192

**Published:** 2013-04-23

**Authors:** Praveen Buddiga, Malik Baz

**Affiliations:** 1 University of California, San Francisco, Baz Allergy, Asthma & Sinus Center, Fresno, CA, USA

## Background

Oxaliplatin is a platinum based agent that is a antineoplastic used in colorectal carcinoma in combination with flourouracil and leucovorin. A 45 year old female patient developed anaphylaxis on prior 2 cycles to this platinum agent with dyspnea and hypotension on the latter cycle. Further cycles were stopped and an allergist consult was obtained.

## Method

In this case a premedication protocol of dexamethasone 10 mg + diphenhydramine 50 mg + famotidine 20 mg by mouth were administered 13 hours, 6 hours and 1 hour respectively prior to the infusion of 40 mg of oxaliplatin in 1:10000, 1:1000, 1:100, 1:10 graded dilutions. Each dilution was infused over 1 hour under close telemetry monitoring. A final dilution that contained 90 % of the dose was infused over 4 hours until completion.

## Results

The patient tolerated the desensitization protocol well with the premedication of dexamethasone+diphenhydramine+famotidine. The graded dilutional desensitization approach allowed the patient to achieve tolerance to the drug without any significant adverse events and was able to continue her cycles of antineoplastic regimen.

## Conclusions

This graded desensitization protocol permits the continuation of antineoplastic treatment protocols in patients that may have a varying degrees of hypersensitivity to oxaliplatin agents.

A premedicated patient in addition to a graded drug dilution approach may allow administration of this potential life saving drug. The patient must at all times during the protocol be closely monitored for any treatment adjustments for tolerability.

